# Editorial: Human-like robotic hands for biomedical applications and beyond

**DOI:** 10.3389/frobt.2024.1414971

**Published:** 2024-05-13

**Authors:** Emanuele Lindo Secco, Yohan Noh

**Affiliations:** ^1^ Robotics Lab, School of Mathematics, Computer Science and Engineering, Liverpool Hope University, Liverpool, United Kingdom; ^2^ Department of Mechanical and Aerospace Engineering, Brunel University, London, United Kingdom

**Keywords:** prosthetic hands, robotic hands, low-cost design, biologically inspired design, human hand

Robotic hands that integrate tactile or force/torque sensors have been utilized to assist robots and amputees in interacting closely with environments and objects. For example, robotic hands have been employed for stable grasping and dexterous manipulation of objects without dropping and damaging them as humans can. Robotic hands have been used for robots and amputees to express their gestures to humans for better communication; in addition, robotic hands fused with tactile or force/torque sensors can make the impossible possible for amputees, such as playing musical instruments like a piano, a guitar, and more just like humans play them while controlling delicate finger forces.

These are common everyday actions that we sometimes take for granted, but they are not. In many situations, these interactions are no longer possible.

With this Research Topic, we have attempted to collect current contributions on the design and integration of novel solutions for the advancement of robotic and prosthetic hands and sensing technology for measurable force, distance, force, etc., which is integrated into the hands.

This Research Topic is the result of contributions from 27 authors and 14 reviewers and editors from 11 countries (Australia, China, Germany, Italy, Japan, the Netherlands, New Zealand, Poland, Switzerland, United Kingdom, and the United States) from 25 Medical Institutions, Academic Institutions and Research Centers (Academy of Physical Education in Katowice, Boise State University, Brunel University, Delft University of Technology, Florida Atlantic University, Georgia Institute of Technology, German Aerospace Center (DLR), Italian Institute of Technology (IIT), Liverpool Hope University, New Mexico State University, Northeastern University, Scuola Superiore Sant’Anna (SSSA), Spaulding Rehabilitation Hospital, Swansea University, Swiss Federal Institute of Technology Lausanne (EPFL), The University of Electro-Communications, Universita’ Campus Bio-Medico, University of Auckland, University of California, University of Florida College of Medicine, University of Naples Federico II, University of Siena, University of Wollongong, Virginia Tech and Worcester Polytechnic Institute).

Here is a brief summary of the main contributions with a link to the details of each study: GP Kontoudis et al. presented An Adaptive Actuation Mechanism for Anthropomorphic Robot Hands where they proposed a novel tendon-driven actuation mechanism for anthropomorphic robotic hands. W Friedl and MA Roa reported a compliant low-cost antagonist servo hand in CLASH—A Compliant Sensorized Hand for Handling Delicate Objects which is an interesting design that provides variable stiffness to the device when grasping everyday objects. C Bosio and colleagues focused on the Scalable Fabrication and Actuation of a Human-Inspired Hand Through 3D-printed Flexures and Combinatorial Actuation: the authors designed fingers that can be manufactured as a single piece and are also inherently compliant. M Lin et al. looked at how rehabilitative treatment of the hand could be improved by means of a soft exoskeleton in combination with playing musical instruments. Details of their work are reported in the paper Feeling the beat: a smart hand exoskeleton for learning to play musical instruments. Finally, M Zandigohar and colleagues analyzed the Multimodal fusion of EMG and vision for human grasp intent inference in prosthetic hand control and consolidated evidence for the importance of using multi-sensory channels in motor learning.

We believe that such a variety of authors and contributions is a clear sign of the urgency and importance of this Research Topic. We hope that this Research Topic will pique the readers’ interest, encouraging them to contact us and these amazing authors for further collaboration and development on this Research Topic.

